# Malnutrition and poverty in India: does the use of public distribution system matter?

**DOI:** 10.1186/s40795-020-00369-0

**Published:** 2020-10-01

**Authors:** Basant Kumar Panda, Sanjay K. Mohanty, Itishree Nayak, Vishal Dev Shastri, S. V. Subramanian

**Affiliations:** 1 International Institute for Population Sciences, Mumbai, India; 2 Department of fertility studies, International Institute for Population Sciences, Mumbai, India; 3 Senior Advisor, FHI Solutions LLC, Alive & Thrive, # 503-506, 5th Floor, Mohan Dev Building, 13 Tolstoy Marg, New Delhi, 110001 India; 4 Harvard Centre for Population and Development Studies, Harvard T.H. Chan School of Public Health, 9 Bow Street, Cambridge, MA 02138 USA; 5 Department of Social and Behavioural Science, Harvard T.H. Chan School of Public Health, Boston, MA USA

**Keywords:** Poor, Underweight, Stunting, BPL, PDS, India, Welfare card

## Abstract

**Background:**

Large scale public investment in Public Distribution System (PDS) have aimed to reduce poverty and malnutrition in India. The PDS is the largest ever welfare programme which provides subsidised food grain to the poor households. This study attempt to examine the extent of stunting and underweight among the children from poor and non-poor households by use of public distribution system (PDS) in India.

**Methods:**

Data from the National Family and Health Survey-4 (NFHS-4), was used for the analysis. A composite variable based on asset deprivation and possession of welfare card provided under PDS (BPL card), was computed for all households and categorised into four mutually exclusive groups, namely real poor, excluded poor, privileged non-poor and non-poor. Real poor are those economically poor and have a welfare card, excluded poor are those economically poor and do not have welfare card, privileged poor are those economically non-poor but have welfare card, and non-poor are those who are not economically poor and do not have welfare card. Estimates of stunting and underweight were provided by these four categories. Descriptive statistics and logistic regression were used for the analysis.

**Results:**

About half of the children from each real poor and excluded poor, two-fifths among privileged non-poor and less than one-third among non-poor households were stunted in India. Controlling for socio-economic and demographic covariates, the adjusted odds ratio of being stunted among real poor was 1.42 [95% CI: 1.38, 1.46], 1.43 [95% CI: 1.39, 1.47], among excluded poor and 1.15 [95% CI: 1.12, 1.18], among privileged non-poor. The pattern was similar for underweight and held true in most of the states of India.

**Conclusions:**

Undernutrition among children from poor households those excluded from PDS is highest, and it warrants inclusion in PDS. Improving the quality of food grains and widening food basket in PDS is recommended for reduction in level of malnutrition in India.

## Background

Poverty is a proximate determinant of malnutrition that mediates through inadequate dietary intake, lack of medical care, lack of access to sanitation and hygiene and poor environment [1–5]. Reduction of poverty and malnutrition through various welfare measures has been the central focus of developing countries. While the national and local government has been implementing many welfare schemes in the key domain of livelihood, health, nutrition, education for reduction of poverty, the effect of these schemes varied by type of services, outcome variable and country-specific [6–9]. Malnutrition among children is positively associated with poverty and has many adverse short and long term consequences; ill-health, cognitive impairment, childhood mortality in short term and the likelihood of developing the non-communicable diseases in the long term [10–15]. Nutrition sensitive interventions for children are increasingly emphasized in the poverty reduction programme of developing countries. Public policies which are successful in reducing poverty has intrinsically targeted multifaceted approach in reduction of malnutrition [16–20]. Despite these, the global progress in reduction of money metric poverty and malnutrition is slow and largely uneven [21, 22]. An estimated 736 million of people in developing countries are living below poverty line (1.90 US Dollar in Purchasing Power Parity income per day) and 151 million under-five children are stunted in 2018 [23, 24].

A large number of studies across and within the countries suggests the strong and significant association of malnutrition with economic factors. While a limited number of studies use income as an economic measures, many studies used consumption expenditure and asset-based index (henceforth refer as wealth index) in explaining malnutrition [25–29]. Cross-country studies found that the gross domestic products (GDP) per capita is negatively associated with malnutrition [30, 31]. Studies also link the absolute income with malnutrition due to unavailability of direct income data in many lower-middle-income countries [31]. Households consumption expenditure, especially food expenditure, is positively associated with the reduction of malnutrition among children and adults [32–34].

A growing number of studies also link malnutrition with asset-based measures using data from demographic health surveys (DHSs). These studies evident that, despite the declination of inequality across the socioeconomic groups, the level of malnutrition is still higher for the poor and disadvantaged and holds true across the countries [22, 28, 29, 35]. Other than economic factors, many non-economic factors such as social identity, household environment, water and sanitation are considered as significant predictors of child malnutrition at household level [3, 35–37]. The maternal characteristics such as maternal education, anthropometry, mothers’ hygienic practice and individual characteristics such as child’s sex, birth order, immunisation, and birth weight are factors associated with malnutrition [25, 36, 38–42].

Poverty estimation and identification of beneficiaries for the welfare programme in India are carried out independently. Poverty estimation at the state level is carried out by the Government of India using consumption expenditure data collected by the National Sample Survey (NSS) on a regular interval. But the households are identified as poor using an independent survey carried out by the state government with central guidelines. The poor households are given a Below Poverty Line (BPL) card that entitled the households to benefits from various welfare schemes of central and state government. The first BPL survey in India was carried out in 1992, and four rounds of the BPL surveys have been carried out since then (Latest in 2011–12) (http://pib.nic.in/newsite-/PrintRelease.aspx?relid=72217). The criteria adopted in each of the rounds to identify the beneficiaries were not uniform. The BPL survey conducted in 1992 used the household income limit of Rs 11,000 annually in classifying poor. The 1997 round of survey used the exclusion and inclusion criterion. In 2002 BPL survey, a set of 13 socio-economic variables were used to compute the wellbeing of the households and identifying the poor. Recent BPL survey adopted three-step methods recommended by Saxena committee report [43]. The BPL beneficiaries are categorised using a 0–10 scoring method based on a weighted score of a set of variable [43, 44]. Studies suggest that a section of the poorer households are excluded from the BPL card [45]. Such exclusion has been attributed to errors of inclusion and exclusion criteria, political influence and corruption [44–46].

The BPL households have long been accorded priority in the welfare programme of central, state and local government in India. Initially, the BPL beneficiaries were provided free/ highly subsidized ration through the Public Distribution System (PDS). However, it has now been extended to a wider domain of goods and services; housing, cooking fuel, health insurance, employment, sanitation, old age pension etc. The benefits are rolled through a number of welfare schemes such as *Pradhan Mantri Awas Yojana, Swachh Bharat Mission, Janani Suraksha Yojana (JSY), Ayushman Bharat Yojana, PM Ujjwala scheme* etc. For example, under the *Swachh Bharat Mission*, the Government of India provided 9.2 crore toilet to the poor households. Recently, the Govt. of India announced the *Ayushman Bharat Scheme*, a health insurance scheme for protecting poorest 40% of the households with coverage up to five lakh rupees for hospitalization (https://www.india.gov.in/spotlight/ayushman-bharat-national-health-protection-mission). We termed the BPL card as a ***welfare card*** that accounts not only free/subsidized ration but also other benefits. Thus, availing the welfare card entitled a poor household to multiple benefits that has a direct and indirect bearing on reduction of malnutrition in India.

The provisioning of subsidized ration through welfare card aims to ensure food availability to the poor households. We conceptualise the paper with the assumption that, the welfare card entitled a household to subsidized ration, other free/subsidized goods and services that have a direct association on the reduction of malnutrition of children in households. However, if a household is excluded from the welfare programme (not having a welfare card), they are more likely to be vulnerable. Though studies have identified the factors affecting malnutrition and poverty, there has been no study on the association of poverty, distribution of welfare card and the nutritional outcome. This paper aims to examine the association of asset deprivation, welfare card with stunting and underweight among children in India. Thus, children belonging to poor households but did not get benefit from welfare schemes are likely to remain at a high level of malnutrition.

## Methods

### Data

The unit data from fourth round of National Family Health Survey (NFHS 4) was used for the analysis. The NFHS 4 is a nationwide cross-sectional household survey conducted with a representative sample of 601,509 households throughout the country during 2015–16. The survey was conducted by the International Institute for Population Sciences (IIPS) with stewardship of Ministry of Health and Family Welfare (MoHFW), Govt. of India (GOI), and technical support from ICF international. The primary objective of the survey was to collect comprehensive information on socio-economic, demographic, health, and nutritional status of mothers and children in the households. The detailed sampling methods, design of the study and findings are available in the national report [47].

The unit of the analysis for this study is the children below 5 year age. Out of the 601,509 households, 164,664 households have at least one children in the selected age-group. A total of 225,002 children under 5 years of age who have the information on height and weight form the final sample of the analysis.

### Outcome variables

Stunting and underweight among children below 5 year are two dependent variables used in the analysis. Stunting is a chronic measure of malnutrition, while underweight indicates both chronic as well as an acute form of undernourishment. In NFHS, the anthropometric information such as height, weight and age in month of the child were recorded. This information was used to calculated the height-for-age Z-scores (HAZ), weight-for-age Z-scores (WAZ), weight-for-height Z-scores (WHZ) using the standardized age and sex-specific growth reference developed by the World Health Organisation (WHO). These z scores are ranged between -6SD to +6SD. If the Z score, falls above or below the limit, it was dropped. If the height for age Z score falls between -6SD to -2SD, the child is classified as stunted. Similarly, if the weight for age Z score (WAZ) falls between the -6SD to -2SD, the child is classified as underweight.

#### Independent variables

The main predictor variable of the analysis, is a composite variable based on the poverty level of the household and possession of welfare card. The NFHS like other DHSs, does not collect data on income or consumption expenditure of the household. To present the economic status of the households, the asset-based wealth index was largely being used in these surveys. The wealth index factor score (WIFS) was computed based on a set of 31 variables in the domain of consumer durables, basic household amenities, housing quality and land ownership of the households using the principal component analysis (PCA). These WIFS are categorised into five mutually exclusive categories named poorest, poorer, middle, richer and richest. These categories are widely used in the literature to estimates the different economic group and to understand economic inequality across and within countries. The national wealth index in NFHS does not account for interstate variations in wealth possession as well as rural-urban differences within states, which lead to biased outcomes. In our study, we had recomputed the WIFS using the principal component analysis (PCA) separately for all the states by place of residence. The wealth index factor score was divided into the 100 percentile for each state and place of residence. The estimates of monetary poverty were available from the *Rangarajan Committee* report for each state and by place of residence. The detailed estimate of the poverty for each state and place of residence is provided in **Appendix 1**. The variable of asset deprived (similar to poverty) is derived under the assumption that the distribution in WIFS and the distribution of consumption poverty are similar across the household. For example, in Maharashtra, 23% households in rural areas and 17% households in urban areas are consumption poor based on the *Rangarajan Committee report.* So, the bottom 23% of distribution of wealth index in rural areas and 17% of distribution of wealth index in urban areas are labelled as asset poor in the state. The household poverty, along with possession of welfare card, was used to compute a composite variable of poverty and beneficiaries of welfare schemes. All households are classified into four groups, and their classification is given below.

#### Real poor

Households categorised as poor based on asset deprivation and having welfare card.

#### Excluded poor

Households categorised as poor based on asset deprivation and do not have welfare card.

#### Privileged non-poor

Households categorised as asset non-deprived and have welfare card.

#### Non-poor

Households categorised as asset non-deprived and not having welfare card.

A set of individual maternal and household level factors are used as independent variables based on the literature review. The individual factors include age (in months), sex, birth order and size of birth of the child. The size of the birth was categorised as very small, small, average and large based on the mother’s response. Similarly, the maternal factors include mother’s education (no education, primary, secondary, higher) and the body mass index (BMI). The BMI of mothers is calculated from the height and weight of the mother and categorised as thin (BMI < 18.5), normal (18.5 < BMI < 25), overweight (BMI > =25). Apart from these the household level factors include caste (SC, ST, OBC and Other), religion (Hindu, Muslim and Other), and place of residence (Rural and Urban) were included as predictor variables in the analysis.

### Statistical analysis

The logistic regression analysis was used to estimate the association of the composite variable of poverty and welfare card on stunting and underweight at national as well as state level. The logistic regression model is one of the widely used statistical techniques for the binary dependent variable which provide the valid and reliable regression coefficient adjusting for the study design and confounder. Stunting and underweight were the binary variables used as outcome variables in the study. The general equation of the logistic model is provided below.

$$ \mathrm{Iogit}\ \left({\mathrm{P}}_{\mathrm{i}}\right)=\upalpha +{\upbeta}_1\left(\mathrm{poverty}\_{\mathrm{wc}}_{\mathrm{i}}\right)+\kern0.5em {\upbeta}_2\left({\mathrm{age}}_{\mathrm{i}}\right)+{\upbeta}_3\left({\mathrm{sex}}_{\mathrm{i}}\right)+{\upbeta}_4\left({\mathrm{bord}}_{\mathrm{i}}\right)+{\upbeta}_5\left({\mathrm{bsize}}_{\mathrm{i}}\right)+{\upbeta}_6\ \left({\mathrm{e}\mathrm{du}}_{\mathrm{i}}\right)+{\upbeta}_7\left({\mathrm{bmi}}_{\mathrm{i}}\right)+{\upbeta}_8\Big({\mathrm{caste}}_{\mathrm{i}\Big)}+{\upbeta}_9\left({\mathrm{rel}}_{\mathrm{i}}\right)+{\upbeta}_{10}\left({\mathrm{por}}_{\mathrm{i}}\right)+{\mathrm{e}}_{\mathrm{i}} $$

where α is the intercept, poverty_wc refers to the composite variable of the poverty and availability of the welfare card in the household. Age, sex, bord, bsize refers to age, sex, birth order and birth size of the child, respectively. Similarly, edu refers to mothers education while bmi is the categorised body mass index of the mother. Moreover, caste refers to caste of household, rel refers to the religion of the household, por includes place of residence. 

The results of the logistic regression was presented as the adjusted odds ratio (AOR), as it is controlled for all other confounding variables. The AOR is here defined as the ratio of the odds of being stunted in the reference category to the odds of being stunted in the predictor category controlled for the other covariates. The Intraclass correlation coefficient (ICC) and the variance inflation factor (VIF) was calculated to understand the multicollinearity among the independent variables. The Pearson chi-square, as well as F-adjusted test statistic was used to test the goodness of fit of the model. The whole analysis was carried out using the STATA 15 software [48].

## Results

Table 1 presents the sociodemographic characteristics of the households (who had at least one child under 5 year age) by the composite variable based on the asset deprivation and availability of welfare card in India. The mean age of the head of household by type of asset deprivation and welfare card is similar. The mean number of children in households are also similar across the four groups. A large proportion of real and excluded poor lives in rural areas. Out of the total households, 57% of real poor and excluded poor households have at least one stunted children while 43% among privileged non-poor households and 36% among the non-poor households. Similarly, the higher proportion of real poor and excluded poor households have at least one underweight children.

**Table 1:** Sociodemographic profile of households by welfare card and asset poor in India, 2015–16

Variables	Real Poor (Asset deprived and have welfare card)	Excluded Poor (Asset deprived and do not have welfare card	Privileged Non-Poor (Asset non-deprived and have welfare card	Non-Poor (Asset non-deprived and do not have welfare card	Total
Mean age of head of household	43.35	38.64	46.42	45.63	44.34
Mean household size	6.20	5.60	6.51	6.18	6.15
Mean number of children in household	1.56	1.59	1.49	1.44	1.50
Percentage of households living in urban areas	6.32	10.50	28.20	42.84	28.73
Percentage of households had a stunted child	56.88	56.95	42.86	35.89	44.06
Percentage of households had an underweight child	54.76	53.46	40.11	33.28	41.35

The state-specific variation in the percentage of households (who had at least one eligible child) by the composite variable based on the asset deprivation and availability of welfare card in India was presented in Table 2. At the national level, an estimated 15% of the households is classified as real poor, 16% as excluded poor, 23% as privileged non-poor and 46% as non-poor. The distribution of households by type of poverty varies enormously across the states of India. For example, in Punjab, 1% of households are categorised as real poor while it was 30% in Bihar. Similarly, 1% of households in Telangana are categorised as excluded poor while it was 37% in Manipur. One of the interesting patterns was observed in terms of privileged non-poor in India. The privileged non-poor avail the welfare schemes that are not meant for them. The pattern shows two south Indian states Andhra Pradesh and Telangana which have more than 70% of the non-poor household had welfare card. Apart from that Kerala, Karnataka and Chhattisgarh possess a higher share of household with a non-poor category having welfare cards. The non-poor who did not have the welfare card is highest in Punjab and Tamil Nadu.

**Table 2:** Percent distribution of households by welfare card and asset poor in states of India, 2015–16

States	Real Poor (Asset deprived and have welfare card)	Excluded Poor (Asset deprived and do not have welfare card	Privileged Non-Poor (Asset non-deprived and have welfare card	Non-Poor (Asset non-deprived and do not have welfare card	Total Households
Telangana	8.68	0.90	71.91	18.51	1504
Andhra Pradesh	9.36	1.46	74.27	14.91	1901
Karnataka	14.20	3.66	56.22	25.92	4672
Kerala	4.22	3.66	26.21	65.91	1855
Chhattisgarh	40.66	5.37	37.07	16.90	6016
Jammu & Kashmir	9.57	7.00	28.40	55.02	5404
Punjab	0.90	7.44	10.49	81.16	3667
Himachal Pradesh	3.72	7.45	19.56	69.27	1935
Uttarakhand	5.87	8.16	22.34	63.63	3728
Nagaland	2.09	8.96	20.08	68.88	2721
Haryana	3.81	9.73	17.88	68.57	4883
Maharashtra	8.38	12.09	20.02	59.52	5882
Gujarat	17.27	14.66	18.97	49.11	4777
Bihar	29.61	15.07	27.44	27.87	14,612
Rajasthan	7.77	15.30	13.79	63.15	10,367
Tamil Nadu	3.47	15.83	7.85	72.85	5372
Mizoram	15.44	16.34	11.10	57.13	3276
West Bengal	15.14	16.73	20.04	48.09	3967
Tripura	10.22	17.55	21.40	50.83	1041
Madhya Pradesh	25.60	19.41	20.69	34.30	15,054
Arunachal Pradesh	22.72	21.35	22.17	33.76	3131
Jharkhand	23.95	21.58	22.02	32.45	7669
Odisha	21.53	24.01	17.22	37.23	8001
Meghalaya	6.98	26.18	12.12	54.72	2744
Assam	21.05	26.77	18.96	33.23	7335
Uttar Pradesh	11.13	27.49	12.19	49.18	24,981
Manipur	9.66	37.29	10.03	43.02	4110
India	15.00	16.13	23.30	45.57	164,664

*The estimates for states and UTs with sample size less than 1000 are not shown in the above table but included for the India estimates

The state-specific estimates of stunting and underweight among the children below 5 year age by the composite variable based on the asset deprivation and availability of welfare card of households were presented in Table 3. The states are arranged in the ascending order of stunting among the excluded poor household. This pattern provides a clear distinction in estimated stunting and underweight among the poor and rich households. About half of the children from real poor and excluded poor households were stunted compared to 37% children from privileged non-poor households and 35% children from the non-poor household. The pattern was similar with respect to underweight. An estimated 48% of children from real poor household, 47% from an excluded poor household, 35% from privileged non-poor household and 29% from the non-poor household were underweight. The pattern shows the vulnerability of both real poor and excluded poor household in terms of stunting and underweight. Interstate variation of the prevalence of stunting and underweight showed a mixed result. In 16 states, the extent of stunting among children of excluded poor households was higher as compared to real poor households. The higher gap among these groups were found in states of Himachal Pradesh followed by Tripura. In 12 states, the extent of stunting among real poor households was found higher as compared to excluded poor household. These includes the states like Telangana, Chhattisgarh and Andhra Pradesh, which had a higher prevalence of welfare cards. In 14 states, the extent of underweight children among real poor households was higher compared to the excluded poor household. Similarly, the prevalence of stunting and underweight were lower in the privileged non-poor as compared to real poor and excluded poor households.

**Table 3:** Percentage of children stunted and underweight by welfare card and asset poverty in states of India, 2015–16

States	Percentage of children stunted				Percentage of children underweight			
	Real Poor (Asset deprived and have welfare card)	Excluded Poor (Asset deprived and do not have welfare card	Privileged Non-Poor (Asset non-deprived and have welfare card	Non-Poor (Asset non-deprived and do not have welfare card	Real Poor (Asset deprived and have welfare card)	Excluded Poor (Asset deprived and do not have welfare card	Privileged Non-Poor (Asset non-deprived and have welfare card	Non-Poor (Asset non-deprived and do not have welfare card
Kerala	24.90	24.25	23.26	18.25	22.32	25.54	19.84	14.08
Telangana	46.34	25.56	28.00	19.66	47.70	37.22	28.48	18.65
Chhattisgarh	43.30	32.74	36.54	29.11	45.34	41.66	34.36	28.44
Andhra Pradesh	42.36	33.88	31.38	27.79	42.35	50.65	31.65	27.99
Arunachal Pradesh	34.68	34.71	25.22	25.16	26.34	27.10	14.64	13.34
Sikkim	41.49	34.97	30.44	24.77	23.46	12.06	15.60	12.05
Tamil Nadu	34.71	35.46	30.56	24.85	35.00	31.64	23.34	22.15
Manipur	32.85	35.67	27.43	22.40	18.10	16.99	13.98	10.20
Tripura	32.01	37.99	19.87	19.33	29.71	34.71	25.33	18.80
Jammu and Kashmir	37.19	39.30	30.04	23.48	26.15	28.07	16.25	14.02
Nagaland	31.02	39.82	28.96	27.12	24.07	25.01	16.56	15.73
West Bengal	39.84	40.04	33.32	27.97	39.68	45.33	28.29	26.36
Punjab	42.93	40.47	24.37	24.63	30.11	35.50	23.00	20.15
Mizoram	36.94	41.15	27.48	21.83	16.39	18.85	13.97	8.31
Odisha	44.28	43.56	32.17	23.11	45.75	43.87	31.30	23.09
Uttarakhand	42.33	44.01	35.95	31.28	36.05	38.24	30.08	23.57
Himachal Pradesh	35.89	45.02	29.11	23.29	27.68	29.86	24.95	19.94
Maharashtra	44.75	45.09	36.89	29.70	51.32	49.64	35.36	31.31
Gujarat	51.23	46.56	40.48	29.96	51.95	49.09	40.65	31.31
Assam	42.07	46.74	31.37	26.94	35.19	40.43	23.12	21.74
Madhya Pradesh	48.17	47.94	39.91	35.29	50.89	48.61	40.91	35.03
Rajasthan	52.53	48.58	38.71	35.59	55.34	47.80	37.22	31.51
Karnataka	48.33	48.63	36.23	28.76	47.84	47.34	35.05	27.62
Haryana	50.38	49.48	37.02	30.40	41.11	38.57	33.80	26.68
Meghalaya	49.51	50.87	47.96	38.85	35.75	35.20	29.78	25.22
Jharkhand	52.84	53.20	45.25	36.35	56.34	55.24	46.50	38.72
Bihar	57.36	56.27	46.83	37.34	50.92	52.00	42.35	34.89
Uttar Pradesh	55.40	57.16	44.63	39.12	47.05	48.63	37.29	34.08
**India**	**49.79**	**49.65**	**37.16**	**31.51**	**47.73**	**46.51**	**34.54**	**29.00**

*Estimates for states with sample size less than 1000 are not shown the above table but included for the India estimates

Table 4 presents the estimates of stunting and underweight by poverty level and sociodemographic characteristics in India. Both stunting and underweight among the children from the real poor and excluded poor households were higher than that of non-poor households. The levels of stunting and underweight were higher among children of higher birth-order, lower birth size, the mother with lower education level and lower anthropometry. For example, among the excluded poor households, 54% of children of mothers with no education were stunted compared to 36% among mothers with 12 or more year of schooling. Similarly, among real poor households, 54% of children among mother with no education were stunted compared to 34% among mothers with 12 or more year of schooling. A similar pattern was also observed for the other categories. But the gap in estimates of stunting and underweight among the well off and worst off varies across the categories and among the variables. For example, for the mothers education, the gap among the mothers who had no education and highest education is higher among the privileged non-poor households (26%) followed by non-poor households (25%), real poor households (20%) and excluded poor households (18%). A similar pattern was also observed for the level of underweight. The gap among the mothers who had no education and highest education among the privileged non-poor and non-poor households was 22% while it is 21% among excluded poor households and 10% among real poor households. This pattern also remains consistent for all other variables such as birth order, size at birth, mothers BMI category and caste of the households.

**Table 4:** Percentage of children stunted and underweight by type of poverty and background characteristics in India, 2015–16

Socio demographic variables	Percentage of children stunted				Percentage of children underweight				Total Number of children)
	Real Poor (Asset deprived and have welfare card)	Excluded Poor (Asset deprived and do not have welfare card	Privileged Non-Poor (Asset non-deprived and have welfare card	Non-Poor (Asset non-deprived and do not have welfare card	Real Poor (Asset deprived and have welfare card)	Excluded Poor (Asset deprived and do not have welfare card	Privileged Non-Poor (Asset non-deprived and have welfare card	Non-Poor (Asset non-deprived and do not have welfare card	
**Age in Month**									
< 12	27.30	27.28	20.03	18.91	37.67	36.20	25.40	23.31	41,871
12–24	53.91	54.42	42.12	36.04	50.18	47.24	34.24	27.56	48,967
> 25	55.31	54.41	41.09	33.61	49.99	49.22	37.71	31.26	1,34,164
**Birth Order**									
1	46.43	45.80	33.24	27.75	45.70	44.31	31.63	25.89	83,046
2–3	48.95	49.01	38.45	32.74	46.63	46.09	35.20	30.03	1,06,012
4+	55.32	55.46	46.97	43.64	52.21	49.94	43.13	38.93	35,944
**Size at Birth**									
Very small	64.04	60.24	50.16	46.39	64.28	59.68	51.66	47.71	6178
Small	56.20	54.05	43.29	38.02	55.62	53.87	42.93	37.49	20,046
Average and above	48.48	48.74	36.24	30.47	46.17	45.15	33.29	27.65	1,98,778
**Sex**									
Female	49.22	49.16	35.97	31.05	47.11	46.43	33.31	28.71	1,16,360
Male	50.33	50.12	38.28	31.93	48.33	46.59	35.68	29.26	1,08,642
**Mother Education**									
No Education	54.28	54.37	48.30	45.15	50.91	50.66	44.03	40.68	68,978
Primary	47.24	48.59	41.58	40.50	46.18	45.07	38.46	35.82	32,835
Secondary	42.15	42.06	33.58	29.60	42.04	40.44	31.49	27.97	1,02,191
Higher	34.01	36.62	22.69	20.08	40.26	29.60	21.85	17.92	20,998
**Place of Residence**									
Urban	47.56	46.12	33.10	28.33	42.00	44.79	31.56	26.73	53,483
Rural	49.93	50.08	38.72	33.78	48.12	46.72	35.68	30.61	1,71,519
**Caste**									
Scheduled Caste	51.94	50.96	40.08	35.65	48.68	47.21	36.95	31.89	42,540
Scheduled Tribe	48.79	48.72	39.88	35.32	51.44	50.14	38.60	35.94	44,440
OBC	50.42	50.22	37.89	32.70	46.77	45.80	35.40	29.99	88,803
Others	45.87	47.12	31.57	26.47	41.79	44.58	29.04	24.90	39,399
**Religion**									
Hindu	50.11	49.48	37.22	31.04	48.62	46.83	34.91	28.98	1,63,089
Muslim	49.15	51.70	37.82	35.28	43.28	45.89	33.22	30.90	35,241
Others	42.92	45.08	33.19	26.36	40.58	42.74	32.84	23.68	26,672
**BMI**									
Underweight	52.96	54.16	43.29	38.30	56.21	56.14	44.36	40.40	53,286
Normal	48.49	48.04	36.67	32.13	43.99	42.58	33.17	28.99	1,39,618
Overweight	41.59	41.77	29.24	24.00	31.71	33.64	24.13	19.30	31,530
**Number of children in Household**									
1	47.82	47.82	34.46	28.73	47.46	45.80	33.06	26.99	113,566
2	51.70	51.31	38.90	34.37	48.06	47.19	35.27	31.27	86,872
3 or more	50.81	50.88	42.61	36.55	47.69	46.84	38.15	31.94	24,564

To find the exposition of malnutrition through poverty status across the household in India and states, multivariate logistic regression method was used. Table 5 presents the adjusted odds ratio (AOR) (with 95% confidence interval) of the child being stunted/underweight by type of poverty of households controlling for all other sociodemographic correlates. Our results suggest that poverty level of the household is a major determinant of stunting and underweight among the under-five children. The risk for stunting in India was highest among the children who were belonging to the excluded poor household with an adjusted odds ratio (AOR) 1.43 [95% CI: 1.39, 1.47] than that of the non-poor household. Similarly, the AOR of a child being stunted from the real poor households was found to be 1.42 [95% CI 1.38,1.46] and privileged non-poor households was found to be 1.15 [95% CI: 1.12, 1.18] as compared to non-poor households. Compared to the children from a non-poor household, the children from real poor households had an AOR of 1.46 (95% CI 1.42, 1.50), the children from excluded poor households had an AOR of 1.37 (95% CI 1.33, 1.40), the children from privileged non-poor  households had an AOR of 1.15 (95% CI 1.12,1.18) to be underweight. The children from a mother who had higher education were less likely to be stunted as compared to those with mothers having no schooling (AOR = 0.47, 95% CI: 0.45,0.48). A similar finding was also observed for the underweight among the children in India. Apart from the mother’s education, mothers BMI status, caste, religion and place of residence of household was also significantly determined the stunting and underweight among the children in India. Apart from these characteristics, the birth size of the children significantly contributes to stunting and underweight among the children in India. Large or average-sized babies were less likely to be stunted and underweight as compared to the children who were very small at birth (AOR = 0.60, 95% CI: 0.57,0.64). Moreover, the female child and the children from urban areas were also less likely to be stunted and underweight as compared to their respective counterpart. Table 6 presents the AOR (with 95% confidence interval) of the child being stunted/underweight by type of poverty of households controlling for all other sociodemographic correlates among the states of India. These results are robust with the pattern obtained at the national level. The results clearly indicate that there is a higher likelihood of stunting as well as underweight across the real poor and excluded poor households than that of the non-poor households.

**Table 5:** Odds ratio and 95% CI of socio-demographic correlates of stunting and underweight among underfive children in India 2015–16

Sociodemographic background characteristics	Stunting	Underweight
**Classified Poor**		
Non Poor**®**		
Real Poor	1.42***(1.38,1.46)	1.46***(1.42,1.50)
Excluded Poor	1.43***(1.39,1.47)	1.37***(1.33,1.4)
Privileged Non-Poor	1.15***(1.12,1.18)	1.15***(1.12,1.18)
**Age of the child (in months)**		
< 12**®**		
12—25	2.73***(2.65,2.81)	1.40***(1.36,1.45)
> 25	2.66***(2.59,2.73)	1.64***(1.60,1.69)
**Sex of the child**		
Male**®**		
Female	0.91***(0.89,0.92)	0.93***(0.92,0.95)
**Birth Order**		
1**®**		
2—3	1.10***(1.08,1.12)	1.10***(1.08,1.13)
4+	1.31***(1.27,1.35)	1.23***(1.19,1.26)
**Size at Birth**		
Average and above**®**		
Small	0.78***(0.74,0.83)	0.75***(0.71,0.80)
Very small	0.60***(0.57,0.64)	0.52***(0.49,0.55)
**Mother’s Education**		
No Education**®**		
Primary	0.88***(0.85,0.90)	0.86***(0.84,0.89)
Secondary	0.68***(0.66,0.70)	0.69***(0.67,0.70)
Higher	0.47***(0.45,0.48)	0.47***(0.45,0.49)
**Place of Residence**		
Urban**®**		
Rural	1.08***(1.06,1.11)	0.99 (0.97,1.02)
**Caste**		
Scheduled Caste**®**		
Scheduled Tribe	0.87***(0.85,0.90)	0.95***(0.92,0.98)
OBC	0.90***(0.88,0.92)	0.93***(0.91,0.95)
Other	0.72***(0.70,0.74)	0.73***(0.71,0.76)
**Religion**		
Hindu**®**		
Muslim	1.11***(1.08,1.14)	0.98 (0.95,1.01)
Other	0.82***(0.79,0.85)	0.54***(0.52,0.56)
**BMI of Mother**		
Underweight**®**		
Normal	0.79***(0.78,0.81)	0.60***(0.59,0.61)
Overweight	0.59***(0.57,0.61)	0.38***(0.37,0.39)
**Number of children in Household**		
1**®**		
2	1.15***(1.13,1.18)	1.05***(1.03,1.07)
3 or more	1.23***(1.20,1.27)	1.05***(1.02,1.09)

® Reference category.

*** *p* < 0.01, ** *p* < 0.05, *p < 0.10

**Table 6:** Odds ratios and 95% confidence interval of type of poverty on stunting and underweight among children in states of India, 2015–16

States/Union Territory	Stunting			Underweight		
	Real Poor	Excluded Poor	Privileged Non-Poor	Real Poor	Excluded Poor	Privileged Non-Poor
Andhra Pradesh	1.16 (0.79,1.71)	0.85 (0.42,1.72)	1.00 (0.76,1.31)	1.12 (0.77,1.64)	1.44 (0.73,2.82)	0.98 (0.75,1.28)
Arunachal Pradesh	1.14 (0.92,1.41)	1.21* (0.96,1.52)	0.98 (0.80,1.22)	1.83*** (1.40,2.39)	1.94*** (1.47,2.56)	1.23 (0.94,1.61)
Assam	1.36*** (1.16,1.59)	1.51*** (1.29,1.76)	1.08 (0.92,1.26)	1.11 (0.93,1.33)	1.39*** (1.18,1.64)	0.92 (0.77,1.09)
Bihar	1.63***(1.50,1.77)	1.61***(1.46,1.77)	1.26***(1.17,1.36)	1.35***(1.24,1.46)	1.43***(1.30,1.57)	1.17***(1.09,1.26)
Chhattisgarh	1.33*** (1.12,1.57)	0.86 (0.67,1.10)	1.22** (1.05,1.42)	1.33*** (1.12,1.58)	1.05 (0.82,1.34)	1.11 (0.95,1.30)
Gujarat	1.78*** (1.51,2.12)	1.50*** (1.26,1.79)	1.42*** (1.22,1.66)	1.64*** (1.39,1.94)	1.55*** (1.3,1.84)	1.32*** (1.13,1.53)
Haryana	1.30** (1,1.69)	1.47*** (1.21,1.78)	1.19** (1.03,1.37)	1.09 (0.84,1.41)	1.21* (1.00,1.47)	1.11 (0.96,1.28)
Himachal Pradesh	1.44 (0.95,2.18)	1.92*** (1.39,2.65)	1.21 (0.95,1.54)	1.47* (0.95,2.28)	1.17 (0.82,1.67)	1.31** (1.02,1.69)
Jammu & Kashmir	1.39*** (1.13,1.72)	1.44***(1.16,1.80)	1.19** (1.01,1.41)	1.41***(1.12,1.78)	1.46***(1.14,1.87)	1.07 (0.88,1.31)
Jharkhand	1.34***(1.18,1.53)	1.36*** (1.19,1.54)	1.23*** (1.09,1.38)	1.39*** (1.22,1.58)	1.36*** (1.20,1.54)	1.20***(1.07,1.35)
Karnataka	1.48***(1.21,1.80)	1.55***(1.13,2.12)	1.10 (0.94,1.28)	1.36***(1.11,1.66)	1.72***(1.26,2.36)	1.04 (0.89,1.21)
Kerala	1.48 (0.90,2.41)	1.57*(0.95,2.58)	1.28 (0.99,1.66)	1.49 (0.90,2.47)	1.54 (0.92,2.58)	1.30 (0.99,1.70)
Madhya Pradesh	1.33***(1.21,1.45)	1.34***(1.22,1.47)	1.12** (1.03,1.21)	1.37***(1.25,1.50)	1.32***(1.20,1.44)	1.07*(0.99,1.17)
Maharashtra	1.20** (1.01,1.42)	1.23***(1.05,1.44)	1.16** (1.02,1.31)	1.47***(1.24,1.74)	1.36***(1.17,1.59)	1.16** (1.03,1.32)
Manipur	1.57*** (1.25,1.98)	1.45*** (1.23,1.71)	1.25* (0.97,1.60)	1.74*** (1.31,2.32)	1.33*** (1.07,1.65)	1.20 (0.87,1.66)
Meghalaya	1.67***(1.25,2.23)	1.47***(1.23,1.76)	1.45***(1.16,1.81)	1.79***(1.34,2.39)	1.39***(1.15,1.67)	1.26 (0.99,1.59)
Mizoram	1.58*** (1.28,1.96)	1.44*** (1.17,1.79)	1.18 (0.94,1.48)	1.56*** (1.18,2.07)	1.54*** (1.17,2.03)	1.31* (0.97,1.77)
Nagaland	0.94 (0.57,1.53)	1.16 (0.90,1.49)	1.02 (0.85,1.23)	1.25 (0.73,2.16)	1.18 (0.88,1.58)	1.03 (0.82,1.28)
Delhi	1.61 (0.84,3.09)	1.49*(0.97,2.29)	1.20 (0.82,1.76)	0.84 (0.43,1.67)	1.17 (0.75,1.82)	0.89 (0.59,1.35)
Odisha	1.63***(1.42,1.87)	1.68***(1.47,1.92)	1.32*** (1.15,1.51)	1.58*** (1.38,1.82)	1.51*** (1.32,1.73)	1.19** (1.04,1.36)
Punjab	1.37 (0.8,2.34)	1.20 (0.93,1.54)	0.92 (0.74,1.14)	1.26 (0.72,2.22)	1.25*(0.96,1.61)	1.04 (0.83,1.30)
Rajasthan	1.32***(1.15,1.52)	1.24***(1.12,1.37)	1.01 (0.91,1.12)	1.54***(1.35,1.77)	1.35***(1.22,1.50)	1.07 (0.97,1.18)
Sikkim	1.35 (0.82,2.22)	1.04 (0.64,1.69)	1.21 (0.79,1.85)	2.03** (1.12,3.69)	0.93 (0.49,1.77)	1.39 (0.8,2.40)
Tamil Nadu	1.24 (0.94,1.64)	1.35*** (1.16,1.56)	1.24** (1.01,1.52)	1.36** (1.03,1.80)	1.30***(1.12,1.51)	0.98 (0.79,1.22)
Tripura	1.52 (0.9,2.55)	1.89***(1.20,2.97)	1.01 (0.64,1.58)	1.35 (0.81,2.27)	1.50 (0.95,2.36)	1.20 (0.78,1.83)
Uttar Pradesh	1.37***(1.26,1.48)	1.45***(1.36,1.54)	1.14***(1.06,1.22)	1.21***(1.12,1.31)	1.30***(1.22,1.37)	1.06 (0.98,1.13)
Uttarakhand	1.08 (0.85,1.36)	1.32** (1.06,1.64)	1.06 (0.91,1.24)	1.4***(1.10,1.78)	1.38***(1.10,1.73)	1.21** (1.03,1.42)
West Bengal	1.26** (1.02,1.55)	1.29** (1.05,1.59)	1.19*(0.99,1.44)	1.26** (1.02,1.55)	1.54***(1.25,1.89)	1.00 (0.83,1.22)
Telangana	1.68** (1.09,2.61)	0.87 (0.31,2.46)	1.16 (0.85,1.58)	1.72** (1.12,2.65)	1.83 (0.71,4.68)	1.28 (0.94,1.74)

*Estimates are controlled for Age, sex, birth order, size at birth of the child, mothers education and BMI of the mother, number of children, place of residence, caste and religion of the household

*** *p* < 0.01, ** *p* < .05, * p< 0.10

Non-poor children is the Reference category

## Discussion

Poverty eradication and reduction of malnutrition have been the central feature of public policy in India since independence. The national, state and local government designed several welfare programmes to reduce the extent of poverty and malnutrition (http://pib.nic.in/newsite/PrintRelease.aspx?-relid=113725). While there has been a secular decline in money metric poverty (from 45% in 1993 to 22% in 2011–12), the reduction of malnutrition remained slow and insignificant [22, 47, 49]. Child malnutrition is continued to be a major public health challenge, affecting around two-fifths of the children in India. The level of stunting among children under 5 year of age had declined from 48% in 2005–06 to 38% in 2015–16, and that of underweight children had declined from 46% in 2005–06 to 34% in 2015–16 [47]. The Government of India implemented various welfare policies which have direct and indirect bearing on the reduction of malnutrition [19, 20, 38, 50–52]. As a programme for reduction of poverty, hunger and food insecurity, the central government issues welfare card to poor households to avail subsidised food through the Public Distribution System (PDS) and other benefit ranging from health insurance, free/subsidised housing and sanitation, cooking fuel, old age pension, electricity and direct cash transfer etc. But some of the disadvantaged/poor households did not get the benefit of this safety net programme, which have a direct and indirect impact on food security. In this context, this is the first-ever study that attempts to link the poverty, welfare card and malnutrition in India. The salient findings of the study are as follows.

First, we found large variation in asset deprivation and possession of welfare card in states of India. At the national level, two-fifths of the household have the welfare card while around one-third of the household is asset deprived. Among the households who had at least one children under 5 year age, 15% were asset deprived and had a welfare card, 16% were asset deprived and did not have a welfare card, 23% were asset non-deprived and had a welfare card and 46% were not asset deprived and did not have a welfare card. The state variation in the distribution of asset deprivation and welfare card is large. Though economically poor states of Uttar Pradesh, Bihar, Chhattisgarh, Madhya Pradesh and Jharkhand had a higher proportion of asset deprived and had welfare card, the exclusion of welfare card among asset poor was also large in these states. For example, in states of Uttar Pradesh, around 27% of households were asset deprived and did not have the welfare card while it was 15% in Bihar and 21% in Jharkhand. In a similar line, the states Andhra Pradesh, Telangana and Karnataka, the largest share of the non-poor household had welfare card. This pattern possesses the inference that there are irregularities in the distribution of welfare card across the households. This indicates that the welfare schemes meant for the poor and needy people are not reaching to them.

Second, descriptive analyses suggest the pattern of stunting and underweight by a composite variable of asset deprivation and provision of welfare card is mixed. At the national level, half of the children from real poor and excluded poor households, while 37% of children from privileged non-poor households and 32% of children from non-poor households are stunted. Similarly, 48% children from real poor households, 47% from excluded poor households, 35% of children from privileged non-poor households and 29% of children from non-poor households were underweight. The state variation of stunting and underweight were striking. The estimates of stunting found higher in real poor households in 13 states of India while it was higher in excluded poor households in another 16 states. A similar pattern was also observed for the level of underweight among the children. Mostly in North-Eastern states such as Manipur, Meghalaya, Tripura etc. the excluded poor households had a higher share of stunting as well underweight. This pattern was also perceived in some high focussed states such as Assam, Uttar Pradesh and Uttarakhand.

Third, the effect of the composite variable of asset deprivation on stunting and underweight are strong. Adjusting for the other covariates, the children from the excluded poor households were 43% more likely to be stunted in India. In comparison, it was 41% in real poor households and 15% in the privileged non-poor household as compared to the children from non-poor households. Similarly, children from the excluded poor households were 37% higher likelihood to be underweight, while it was 46% in real poor households and 15% in the privileged non-poor household. The pattern was mixed across the states of India (Table 6). Among the states, Uttar Pradesh, Bihar, Jharkhand and Madhya Pradesh had a significantly higher likely of stunted children in excluded poor households. This pattern also confirmed that higher likely of the children underweight among the real poor households in most of the states.

Fourth, apart from the poverty and welfare card, this study also evident other socioeconomic factors greatly influence nutrition among children in India. Among the other factors, maternal education, anthropometry, caste, religion and place of residence of households have significantly correlated with stunting as well as underweight among the children in India. The observed differential in undernutrition widens the gap in disparity among the poor and rich. The gap in income, education and living standard affects the food security of the households which is main reason behind the increase in the malnutrition in India and other developing countries. Moreover, the children from the higher birth order and lower birth size have significantly associated with the stunting and undernutrition among children in India. These reasons are multifaceted and intertwining affects the undernutrition status of the children in India. There is some explanation given based on the above finding. The unavailability of the welfare card among the poor and availability of welfare card among the richer households are explained as the misuse of welfare card [44–46]. The non-availability of the welfare card makes the household more vulnerable in terms of food grain, employment, and social welfare scheme and health accessibility, which have a combined impact on child malnutrition. Moreover, the availability of welfare cards in the non-poor household makes them privileged non-poor in gaining these facilities. The possibility of poor nutritional outcome among households deprived in asset and having a welfare card are two folds. First, though the PDS focus on making provision ration, the quality of ration is poor. Several studies have raised concern on the quality of food grain under the Public Distribution System (PDS) [53]. A Second plausible explanation is a higher chance of selecting the real poor by two independent approaches. While the asset deprivation is taken by an independent survey which is based on the holding of assets in a household, the BPL is distributed to the poorest based on predefined criterion. What we observed, the real poor and non-poor are two true groups that are correctly defined. However, a section of asset poor and not having a welfare card are another concern. The poverty level among these households is not different from those who are real poor. The lack of access to basic services, illiteracy and exclusion from the welfare scheme in these households are all multi-faceted manifestations of stunting and underweight among the children. Though the Government of India introduced the national food security bill which guaranty food availability for the needy people, it has not adequately addressed these households due to the exclusion of welfare card.

This study has the following policy implication. First, the government should take a strong step in improving nutritious food under the PDS. Provisioning of good quality nutrients under PDS and widening baskets of food grain under PDS may be help reduce malnutrition. Second, households who are poor and excluded from the welfare card should be given priority and included under the welfare schemes. This will help to get universal access to food and improved nutrition and achieving the central goals of development processes aiming reduction of poverty and inequality.

We outline following the limitations of this study. First, we believe that those household have a welfare card do use the card to avail benefits from welfare schemes. Second, we also assume that the relative ranking in wealth index and consumption expenditure used to estimate poverty are similar. Third, the time period of poverty estimates is based on 2011–12, while the welfare card information was taken in 2015–16.

## Conclusion

We found that our estimates of the effects of poverty and not having welfare cards show a positive impact on child malnutrition in India. The study supports that systematic effort is needed to reduce the extent of malnutrition, especially among the poor. These results also support the case for broadening access to universal food security and comprehensive coverage welfare cards for the needy population, which is vital in declining maternal as well as child malnutrition in India.

## Supplementary information

**Additional file 1.**

## Data Availability

The unit-level data is available from the Demographic Health Survey (DHS) data repository through https://dhsprogram.com/data/dataset/India_Standard-DHS_2015.cfm?flag=0/ and could be accessed upon a data request subject to non-profit and academic interest only. In another case, the corresponding author of the paper may be contacted.

## Abbreviations

NSS: National Sample Survey
BPL: Below Poverty Line
JSY: Janani Suraksha Yojana
PDS: Public Distribution System
NFHS: National Family Health Survey
