# Supplementary material for: Malnutrition and poverty in India: does the use of public distribution system matter?
Source: BMC Nutr. 2020 Oct 1;6:41. doi: 10.1186/s40795-020-00369-0 (PMC7528460; doi:10.1186/s40795-020-00369-0)
Supplement: Supplementary file 1 — Additional file 1. [file 40795_2020_369_MOESM1_ESM.docx]

**Appendix-1: Percentage of population classified as consumption poor, households that had Welfare card, stunting and underweight by states of India.**

| State/Ut | Percentage of households have Welfare card | Percentage of population estimated as consumption poor (Rural) | Percentage of population estimated as consumption poor (Urban) | Percentage Households have consumption poor (Total) | Percentage Stunted | Percentage Underweight | Number of household | Number of children |
| --- | --- | --- | --- | --- | --- | --- | --- | --- |
| Andaman and Nicobar Island | 44.88 | 6.60 | 4.90 | 6.00 | 23.31 | 21.32 | 468 | 578 |
| Andhra Pradesh | 40.01 | 12.70 | 15.60 | 13.70 | 31.95 | 32.48 | 1,901 | 2,599 |
| Arunachal Pradesh | 57.06 | 39.30 | 30.90 | 37.40 | 29.5 | 19.68 | 3,131 | 3,851 |
| Assam | 3.81 | 42.00 | 34.20 | 40.90 | 36.49 | 30.06 | 7,335 | 8,855 |
| Bihar | 77.74 | 40.10 | 50.80 | 41.30 | 48.72 | 44.26 | 14,612 | 22,275 |
| Chandigarh | 17.79 | 12.00 | 21.50 | 21.30 | 29.76 | 24.46 | 132 | 174 |
| Chhattisgarh | 7.37 | 49.20 | 43.70 | 47.90 | 37.91 | 38.27 | 6,016 | 8,230 |
| Dadra and Nagar Havel | 18.35 | 55.20 | 15.30 | 35.60 | 41.84 | 39.42 | 219 | 277 |
| Daman and Diu | 36.23 | 0.00 | 17.60 | 13.70 | 23.12 | 27.27 | 260 | 329 |
| Goa | 21.69 | 1.40 | 9.10 | 6.30 | 20.32 | 23.66 | 306 | 378 |
| Gujarat | 23.28 | 31.40 | 22.20 | 27.40 | 38.64 | 39.82 | 4,777 | 6,444 |
| Haryana | 37.98 | 11.00 | 15.30 | 12.50 | 34.32 | 29.76 | 4,883 | 6,875 |
| Himachal Pradesh | 45.98 | 11.10 | 8.80 | 10.90 | 26.66 | 22.03 | 1,935 | 2,525 |
| Jammu and Kashmir | 70.42 | 12.60 | 21.60 | 15.10 | 27.9 | 16.91 | 5,404 | 7,093 |
| Jharkhand | 30.43 | 45.90 | 31.30 | 42.40 | 45.9 | 48.22 | 7,669 | 10,507 |
| Karnataka | 28.65 | 19.80 | 25.10 | 21.90 | 36.6 | 35.53 | 4,672 | 6,308 |
| Kerala | 46.29 | 7.300 | 15.30 | 11.30 | 20.11 | 16.43 | 1,855 | 2,235 |
| Lakshadweep | 28.39 | 0.6 | 7.90 | 6.50 | 27.22 | 23.53 | 219 | 281 |
| Madhya Pradesh | 19.69 | 45.20 | 42.10 | 44.30 | 42.24 | 43.22 | 15,054 | 21,272 |
| Maharashtra | 19.1 | 22.50 | 17.00 | 20.00 | 34.42 | 36.19 | 5,882 | 7,990 |
| Manipur | 26.53 | 34.90 | 73.40 | 46.70 | 29.05 | 13.96 | 4,110 | 5,256 |
| Meghalaya | 22.16 | 26.30 | 16.70 | 24.40 | 44.09 | 29.33 | 2,744 | 3,823 |
| Mizoram | 23.7 | 33.70 | 21.50 | 27.40 | 28.14 | 12.01 | 3,276 | 4,309 |
| Nagaland | 38.75 | 6.100 | 32.10 | 14.00 | 28.81 | 16.97 | 2,721 | 3,825 |
| Delhi | 18.18 | 11.9 | 15.70 | 15.60 | 32.46 | 27.7 | 906 | 1,145 |
| Odisha | 11.4 | 47.80 | 36.30 | 45.90 | 34.34 | 34.59 | 8,001 | 9,728 |
| Puducherry | 21.56 | 5.90 | 8.60 | 7.70 | 23.82 | 22.55 | 744 | 945 |
| Punjab | 30.78 | 7.40 | 17.60 | 11.30 | 26.11 | 21.83 | 3,667 | 4,746 |
| Rajasthan | 11.32 | 21.40 | 22.50 | 21.70 | 39.44 | 36.79 | 10,367 | 14,916 |
| Sikkim | 31.62 | 20.00 | 11.70 | 17.80 | 29.42 | 14.22 | 805 | 898 |
| Tamil Nadu | 23.32 | 24.30 | 20.30 | 22.40 | 27.42 | 24.3 | 5,372 | 6,836 |
| Tripura | 28.21 | 22.50 | 31.30 | 24.90 | 24.21 | 24.23 | 1,041 | 1,188 |
| Uttar Pradesh | 35.18 | 38.10 | 45.70 | 39.80 | 46.58 | 39.92 | 24,981 | 36,465 |
| Uttarakhand | 80.59 | 12.60 | 29.50 | 17.80 | 34.12 | 27.09 | 3,728 | 5,007 |
| West Bengal | 38.3 | 30.10 | 29.00 | 29.70 | 32.91 | 32 | 3,967 | 4,810 |
| Telangana | 80.06 | 12.70 | 15.60 | 37.40 | 27.85 | 28.21 | 1,504 | 2,029 |
| India | 38.37 | 30.90 | 26.40 | 29.50 | 38.66 | 36.09 | 1,64,664 | 2,25,002 |
